# The Maryland Dental Law

**Published:** 1894-02

**Authors:** 


### Editorial, Etc.

THE MARYLAND DENTAL LAW.—When the Maryland Legislature was in session in 1892 we gave in an editorial in the January number of that year the history of the movement that resulted in the passage of the dental law, which was approved March 31, 1884, and the reasons for the amendments thereto by the Legislature of 1886. In 1888 the authorized code of public general laws superseded all the statute laws of the State of Maryland, and the dental law was again changed, in these particulars:

1. The law has no application to persons engaged in the practice of dentistry prior to March 31, 1884.

2. As to all other persons it is unlawful for them to practice without a certificate or diploma.

3. As the law now stands, it is uncertain as to cases occuring prior to the adoption of the Code whether they can get certificates by mere registration, or by examination before the board.

4. That one who is practicing without a certificate must be shown to have wilfully violated the law.

As the existing dental law is not expressed in precise, accurate terms, it has been proposed to have it amended. Bills were drafted and presented to the Legislatures of 1890 and 1892, but failed to reach a vote. A conference committee, composed of three members each from the Maryland State Board of Dental Examiners and of the Maryland State Dental Association, has the matter under consideration at this time. As the subject of dental legislation is of vital and practical importance to all dentists in the State, several features are suggested to be embraced in the scope of the new act, which is intended to supersede the existing dental law.

1. A uniform standard of license should be established, so that if it is proposed to examine the dental graduates of any university or college chartered by the State, all should be included; although in ten years no diploma has been questioned, nor any college published as derelict by the Dental Examiners of Maryland.

A dental college diploma might exempt an applicant from examination, but it should not be made a pre-requisite for examination. This is a rule in examinations for the bar, and it has worked satisfactorily.

2. Upon ordinary principles, in order to harmonize opposing interests in the organization of the new board, equal representation of each and every dental college or department should be recognized, as in Pennsylvania where the dental board is composed of three from the dental colleges and three from the profession at large. The duty of the board will be the protection of the public; the duty of each teacher in the board will be to guard the interests of his school.

3. Only reputable graduates in dentistry should be appointed by the Governor.

4. Examinations should be submitted in writing, and the questions and answers filed to be inspected by candidates and others interested.

5. The favorable vote of three members (if the board consists of five) should be sufficient to grant a permanent certificate. This power is a large one, but it is only fair to candidates whose examinations as to their knowledge and skill in dental surgery prove satisfactory to a majority of the board.

6. Provide that certificates shall be issued by the dental board only, and not by authority of the officers of the board or individual members of the board.

7. Exempt from registration non-resident dentists attending individual cases.

8. Provide for civil action against unlicensed practitioners to recover money paid by patient.

9. Forbid a minor to practice dentistry.

10. Forbid the assumption of the academic title "Doctor" unless conferred.

11. Forbid practicing under a diploma forged or bought.

12. Punish false personation of another practitioner.

Our objections to the bill introduced in 1892 were not on the ground of its policy or constitutionality, but were stated in the following analysis of the bill:

The bill does not emanate from the State board of dental examiners or any incorporated dental body of the city or State, or mass-meeting of dentists. It makes radical changes in the existing dental law, which will be oppressive to *all* the dentists now in practice, requiring them to register again and pay again for new certificates; it makes a dental diploma a prerequisite for examination, whereas, when a young man has studied law and a board of examiners decided as to his competency he is admitted to the bar; it prevents the appointment by the Governor, even though he elect to appoint men of recognized ability and honor, of any practicing dentists whose names are not recommended by a mass-meeting of the dentists of the State, and at such a mass-meeting four years ago only one dentist from the twenty-three counties of the State attended; it provides that the notice for the mass-meeting shall be given by "postal card or otherwise," in the line of Mr. Elkin's famous West Virginia Central by-law, 'at Piedmont or elsewhere,' not through notice in a paper like *The Sun*, but by publication, it may be, ("constructive service") in a paper or dental journal that circulates among the dentists of Maryland from the mountains to the ocean about as much as it does in the moon; it provides that the mass-meeting shall be called in like manner every time there is a vacancy; it provides for the "punishment of any persons who *may have offended*," thus rendering null and void certificates issued prior to the passage of the act; it prohibits a member of the board to serve longer than two consecutive terms, and then, inconsistently; by another section, continues one member of the board in office for *three* consecutive terms, or ten years; it makes no provision for members absenting themselves from meetings (say from two regular meetings consecutively as is customary in dental laws) as should be in the Maryland dental law, because for lack of a quorum but one meeting has been held since July, 1890, no 'annual report' made to Governor Jackson in 1891 or to Governor Brown in 1892, as the existing dental law requires; and it does not provide, as in all cases should be the rule, that the theoretical examination be submitted in writing and the questions and answers filed, as elsewhere there was "witnessed the examination of a student at one time by a gentleman who could not answer the questions himself —questions from a quiz-book."

The true reason for a dental law is that experience has shown the wisdom and policy of securing the community against the impositions of ignorance and quackery, and "of course our dental laws will remove this evil when the present crop of this class will be so kind as to die off." Such laws have been held to be constitutional, and the Supreme Court of the United States has affirmed the right of a State to regulate the practice of professions.

The latest dental law which has gone into effect is that of the State of Connecticut, three sections of which we quote.

SEC. 12. Cruelty, incapacity, unskillfulness, gross negligence, indecent conduct toward patients, or any such professional misbehavior as shows unfitness on the part of the dentist, shall be sufficient cause for the revocation of a license, or prohibition to practice as above provided; and whenever complaint shall be made to any of said commissioners against any dentist practicing in this state, said commissioners shall investigate the matter, and on finding probable cause shall notify the party complained of to appear before them and show cause why he should not be prohibited, or why his license should not be revoked.

SEC. 13. Every such notice shall be in writing, and signed by the recorder, and shall contain a statement of the causes for which such prohibition or revocation is claimed, and shall specify the place and time for the hearing, which shall be at least twelve days after the service of said notice. Said notice may be served by leaving a copy thereof attested by the recorder, at the place of business of the party complained of or at his last usual place of of abode, or by sending the same by mail.

SEC. 14. Any dentist who shall at any hearing before the commissioners, either by himself or by his procurement, make any false statement or misrepresentation with intent to deceive or mislead said commissioners, shall thereby forfeit his license, or be prohibited from practice.

Some of its features have been considered worthy of incorporation into the bill to be presented to the Legislature of 1894. Whether it will invite the criticism of the bill of 1892 of which it was said "the dental bill reaches its insidious fingers back eight years into the past and gives a dental board formed by a fearful and wonderful method the power to close up the office of any dentist who has gone into the profession within the last eight years," will not be known until a committee is "appointed to draw up resolves, declarations of the constitutional rights and privileges of the freemen of the province."

